# Parotid Duct Repair by Facial Vein Graft versus Gore-Tex, A Sialographic Evaluation

**Published:** 2013-06

**Authors:** R Gheisari, C Mohamadinezhad, R Mehravaran, M Ziaei

**Affiliations:** a Dept. of Oral and Maxillofacial Surgery, School of Dentistry, Shiraz University of Medical Sciences, Shiraz Iran.

**Keywords:** Parotid Duct Repair, Sialography, Gore-Tex Tube, Vein Graft

## Abstract

** Statement of Problem: **The most common method for parotid duct anastomosis is suturing. A ductal defect of greater than 1cm may prevent a direct anastomosis.

**Purpose:** The goal of this study was a sialographic evaluation to compare repairing a parotid duct with facial vein graft versus Gore-Tex tub in 19 dogs.

**Material and Methods: **Nineteen dogs were studied in this experimental trial. Extra oral transverse incisions were made in buccal regions bilaterally to expose parotid ducts and a defect (2 cm) was performed in similar areas (right and left). The right resected duct was repaired with facial vein graft and the left anastomosis was performed by using the Gore-Tex tube microscopically. Sialography was used to evaluate the ductal leakage. Statistical analysis was performed, using SPSS software and McNemar’s test.

**Results:** Based on the sialography evaluation; the ductal leakage was seen in five cases (26.31%) on the right side and in seven cases (36.84%) in the left side. Statistical analysis using McNemar’s test suggested no statistically significant difference between ductal leakages in right and left parotid ducts (*p*> 0.05).

**Conclusion: **The results of this study suggest that the efficacies of Gore-Tex tube and vein graft in parotid duct anastomosis are similar, but the use of Gore-Tex tube had a number of advantages, including reduced morbidity of the graft and short operation time.

## Introduction

The parotid gland lies superficial to the posterior aspect of the masseter muscle. The parotid duct (Stenson's duct) is approximately 6 cm in length and 5mm in diameter [1]. 

Sharp penetrating face trauma causes an esthetical defect and may injure deeper anatomic structures such as parotid duct. The parotid duct injury is commonly caused by stab wounds from knives or other sharp objects. Failure to recognize a parotid duct injury may result in the formation of sialoceles, cutaneous fistulas or salivary duct cysts [2]. 

Sialography is the gold standard in radiological evaluation of the salivary glands and the ducts for obstructive, infectious and neoplastic diseases [3]. The chosen method of treatment is based on the age, site and mechanism of the injury. There are generally three methods employed to manage duct lacerations. These treatments include primary repair of the duct with microsurgical anastomosis, diversion of the salivary flow by formation of an oral fistula and suppression of the salivary gland function [4]. 

The most common method of anastomosis is suturing [5]. A ductal defect of greater than 1cm may prevent direct anastomosis, and an autogenous graft may be indicated and an interposition vein graft has been reported to repair the parotid duct [6]. 

The use of alloplastic tubes seems attractive concerning their advantages which include: preventing donor site morbidity, decreased risk of infections, decreased surgical time and easy availability. One of the alloplastic materials that have been used for duct or lumen reconstruction is polytetrafluoroethylene (PTFE). PTFE is considered as a physiologically inert material that can be mechanically expanded. This property is widely used to produce micro porous membranes such as Gore-Tex. [7- 8].

 Therefore, the researchers have focused on the methods with better efficiencies and less complications. In addition, no indication in the published studies supports the efficacy of alloplastic tubes in the parotid duct anastomosis. In this study, the efficacy of Gore-Tex tube in parotid duct anastomosis was compared to the facial vein graft.

## Material and Methods

**Figure 1:**
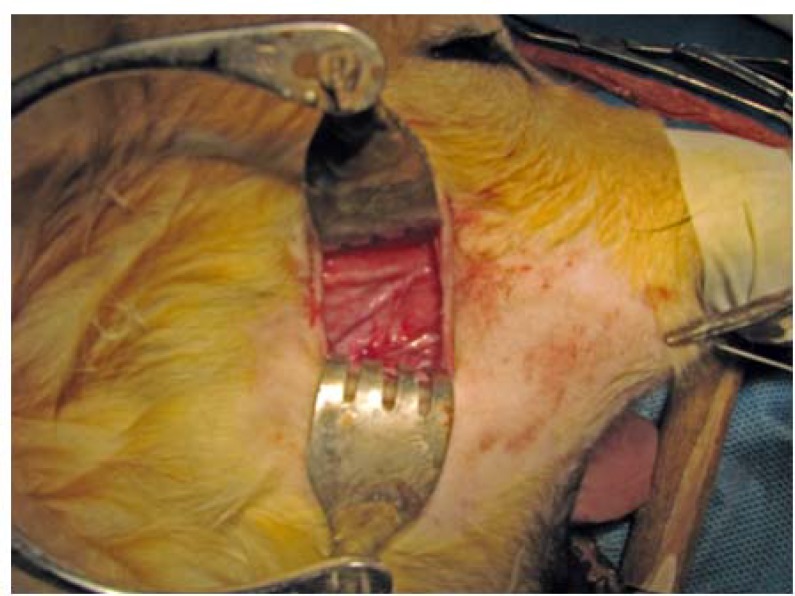
Parotid duct and vein exploration

Nineteen dogs (of the same breed and gender) were studied in this experimental trial. Intravenous Cephalothin (1 g) and intramuscular dexamethasone (8 mg) were administered preoperatively for all cases and local antibiotic was administered postoperatively. Extra oral transverse incisions were made in buccal regions bilaterally to expose parotid ducts (Figure 1). 

**Figure 2a:**
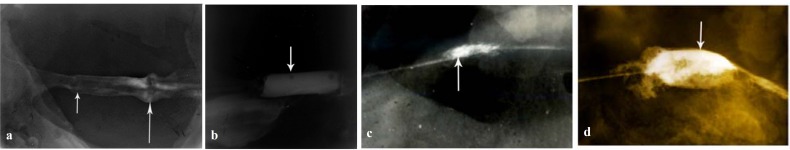
Sample of parotid duct repair by vein graft without leakage      **b **Sample of parotid duct repair by Gore-Tex without leakage      **c **Sample of parotid duct repair by vein graft with leakage      **d **Sample of parotid duct repair by Gore-Tex with leakage

Subsequently, a ductal defect, sized 2cm, was performed in similar areas (right and left). The right resected duct was repaired with right facial vein graft and the left anastomosis was performed, using Gore-Tex tube (GT; W.L.Gore and Associates, flagstaff, AZ). All anastomosis was done by an end-to-end anastomosis technique with 8-0 nylon sutures microscopically. The operation time was measured in all cases for two methods. Twenty one days after the operation, sialography was performed to evaluate the quality of the anastomosis repair and ductal leakage (Figure 2). The parotid ducts were inspected by radiographic examination after the injection of contrast medium (Meglumine; Daroupakhsh, IRAN) into the right and left ducts by a fine intravenous line tube from the duct papillae. Statistical analysis of the obtained data was performed using SPSS version 11.5 (SPSS, Chicago, IL) and the McNemar’s test. The differences with P values less than 0.05 were considered statistically significant.

## Results

Nineteen dogs (with similar race and sex) were studied. Twenty one days after operation, sialography was performed to evaluate the operation outcomes. 

Right and left ductal leakage was found in 5 cases (26.31%) and 7 cases (36.84%) respectively.

It was shown that the duration of parotid duct repair with vein graft was more than anastomosis with tube.

Statistical analysis of the obtained information, using McNemar’s test suggested no statistically significant difference between the ductal leakages in right and left parotid ducts (*p*> 0.05).

## Discussion

There are many studies about the use of prosthetic tubes for vascular defects reconstruction in micro vascular surgery field. These studies have reported a variety of success rate. 

We found ductal leakage in 5 cases using vein grafts and in 7 cases using Gore-Tex anastomosis but, there was no statistically significant difference between two methods (*p*> 0.05). This is probably because of a better healing process of the gap space that is present in the vein - duct anastomosis area. Patency rates over 5 years of the axillary artery repair with PTFE graft ranges from 30 to 80 % [9]. Some authors have reported 57% 5-year patency whereas others have found a high rate of re-infection and low patency which required re-intervention [10].

Kedora and colleagues [11] conducted a prospective randomized study, comparing the PTFE stent grafts with femoro-popliteal bypass. The PTFE stent graft had a 1-year patency rate comparable to the surgical bypass, with a significantly shorter hospital stay [11].

A meta-analysis study suggests unsatisfactory results when PTFE-coated grafts were used for bypassing to the infra-popliteal arteries [12].

Some studies compared the human umbilical vein (HUV) with PTFE and saphenous vein and showed that HUV was better than PTFE but worse than saphenous vein in terms of 5-year patency [13-14].

Although prosthetic grafts are readily available, easy to handle and do not require extensive dissection to harvest; their propensity to undergo thrombosis and develop neo-intimal hyperplasia makes them a less favourable alternative when compared to the vein grafts [15]. 

According to the aforementioned studies, prosthetic graft occlusion may have more severe consequences than the vein graft occlusion. However, if vein is truly unavailable; PTFE would the best option for the vascular bypass.

Ichihara et al. [16] believed that short-distance nerve defects in humans can be successfully treated by alloplastic tubes as an artificial nerve guides; in contrast, Pitta [17] and colleagues have not recommended the use of Gore-Tex tube in trigeminal nerve branches reconstruction because of the poor outcomes. 

## Conclusion

This study suggest that the efficacies of Gore-Tex tube and vein graft in parotid duct anastomosis are similar, but the use of Gore-Tex tube had a number of advantages including reduced graft morbidity and short operation time. Future randomized, controlled experimental and clinical trials with more samples and long follow-up periods are necessary to validate if the PTFE tube is comparable with the autogenous vein for parotid duct repair. 
